# Can Handgrip Strength Improve Following Body Mass-Based Lower Body Exercise?

**DOI:** 10.1089/biores.2017.0008

**Published:** 2017-03-01

**Authors:** Yu Yaginuma, Takashi Abe, Robert S. Thiebaud, Takahiro Kitamura, Masashi Kawanishi, Tetsuo Fukunaga

**Affiliations:** ^1^ Department of Sports and Life Sciences, National Institute of Fitness and Sports in Kanoya, Kagoshima, Japan.; ^2^ Department of Kinesiology, Texas Wesleyan University, Fort Worth, Texas.

**Keywords:** cross education, exercise intensity, maximum isometric strength, muscle thickness

## Abstract

Knee extension strength (KES) improves following body mass-based lower body exercise training; however, it is unknown whether this type of exercise increases handgrip strength (HGS) as a result of a cross-education effect in older individuals. Our aim was to investigate the effect of a body mass-based exercise intervention on HGS and KES in older adults. At baseline, 166 subjects started a 12-week intervention program, and 160 (108 women and 52 men) subjects completed the study. A self-selected group of 37 older adults (21 women and 16 men) served as a control group. HGS, KES, and ultrasound-derived anterior thigh muscle thickness (anterior thigh MT) were measured at baseline and post-testing, and relative strength of the knee extensor (KES/anterior thigh MT) was calculated. A linear regression model controlling for baseline values of body–mass index, % body fat, fat-free mass, HGS, chair stand time, anterior thigh MT, and KES/body mass ratio found a significant difference between control and training groups for KES post-testing values (*p* = 0.001) and anterior thigh MT post-testing values (*p* = 0.012), but not for HGS post-testing values (*p* = 0.287). Our results suggest that increases in lower body strength and muscle size following a 12-week lower body mass-based exercise intervention fail to translate into improvements in HGS.

## Introduction

Age-related alteration in handgrip strength (HGS) is a powerful predictor of future disability^1,2^ and mortality.^3,4^ However, the mechanism explaining the inverse relationship between HGS and morbidity/mortality in middle-aged and older adults is not fully understood. One way to explore this mechanism is to investigate the underlying factors contributing to individual differences in HGS level among middle-aged and older adults, which may be associated with multiple factors such as heredity and environment (e.g., living conditions, physical activity level, and nutritional state). Evidence supports that birth weight is positively correlated with adult HGS.^5,6^ Furthermore, nutritional status changes throughout life are also regarded as an important contributing factor in muscle mass and HGS losses.^7,8^

Whole body resistance training and/or direct handgrip training may also improve HGS in middle-aged and older adults. When resistance exercise is offered using strength training machines, subjects sit on a chair and (in many cases) the subjects' hands grip a bar to maintain body position during the exercise. This type of exercise indicates indirect handgrip exercise.

In a direct effect on forearm/hand muscles, high-intensity handgrip training improved HGS.^9^ Likewise, HGS significantly improved following high-intensity (60–80% of one repetition maximum; 1RM), machine-based, whole body resistance training without handgrip exercise.^10–13^ However, inconsistent results are found in studies using low to moderate intensity, nonmachine-based (e.g., elastic bands), upper and lower body resistance training without handgrip exercise in older adults.^14–18^ The discrepancy between studies may be associated with the modality and/or exercise intensity of the intervention, which includes direct or indirect handgrip training and/or training-induced neural adaptations.

The cross-education effect is an interlimb phenomenon where strength gains are detected not only in the unilateral trained limb but also in the contralateral homologous limb that was not targeted with training.^19^

The increase in strength of the untrained contralateral limb may be associated with the exercise intensity in the trained limb.^20^ One study reported that body mass-based upper and lower body exercise training with and without elastic bands elicited an increase in HGS in older adults.^21^ In that study, direct or indirect handgrip training may not have been involved. Therefore, training-induced neural adaptations may play a role in the training-induced increase in HGS. Although the cross-education effect can occur in both upper and lower limb muscles,^19^ it is unknown whether HGS can improve from resistance training-induced increases in strength of the large upper and lower body muscles.

Home-based lower body exercises such as the squat are commonly incorporated in activities of daily living, and those daily activities seem to be affecting HGS.^21^ Therefore, the associations between HGS and morbidity/mortality may be improved by lower body exercise that includes the squat movement. However, the exercise intensities in the quadriceps during a squat movement are dependent on a ratio of knee extension strength (KES) to body mass. For example, one study found that the estimated intensity during the squat movement was 72% of maximal muscle activation (EMGmax) in frail elderly, 52% EMGmax in older adults, and 25% EMGmax in middle-aged adults.^22^ We hypothesized that body mass-based lower body strength training would improve HGS in older adults who have low knee extensor strength due to the higher relative exercise intensities. Thus, the purpose of this study was to investigate the effect of a lower body mass-based exercise intervention on HGS in older adults who have varied knee extensor/flexor strength.

## Methods

### Subjects

The present study analyzed data from three cohort studies: the Kanoya Chokin Study,^23^ Shibushi Chokin Study, and Bando Chokin Study. A total of 203 older adults (134 women and 69 men) were recruited through a printed advertisement and by word of mouth in the Ohsumi-Kagoshima area. Before making a decision to separate the experimental groups (intervention group or nonexercising control group), all of the subjects (*n* = 203) were interviewed by a researcher. During the interview process, a number of the subjects requested to be participants in the intervention group, and a number of subjects (*n* = 37) self-selected themselves to participate in the control group.

At baseline, 166 subjects started a 12-week intervention program, and 160 (108 women and 52 men) subjects successfully accomplished the entire experiment and completed baseline and post-testing measurements (Table 1). Three subjects dropped out during the study period (for various personal reasons) and another three subjects did not complete post-testing measurements. Therefore, these six subjects were excluded from the study. Before obtaining informed consent, a written description of the purpose of the study and its safety was distributed to potential subjects. The subjects had a medical screening before participation in this study. If subjects did not have this medical screening, their medical condition was assessed by self-report, which was based on annual health examinations or family doctor.

**Table 1.** **Effects of Body Mass-Based Exercise on Body Composition, Physical Function, and Muscular Strength in Older Adults**

	Training (*n* = 160)		Control (*n* = 37)	
Variables	Baseline	12 weeks	Baseline	12 weeks
Age (year)	69 (6)		69 (7)	
Sex (% men)	33		43	
Height (m)	1.55 (0.08)	1.55 (0.08)	1.56 (0.06)	1.56 (0.06)
Body mass (kg)	56.0 (8.7)	55.6 (8.5)	56.2 (8.2)	55.6 (7.6)
Body–mass index (kg/m^2^)	23.2 (2.9)	23.0 (2.8)	22.9 (2.5)	22.7 (2.3)
Body fat (%)	29.2 (7.0)	28.7 (7.0)	28.6 (5.7)	28.4 (6.0)
Fat-free mass (kg)	39.6 (7.0)	39.5 (6.9)	40.0 (6.3)	39.8 (6.2)
AnT-MT (cm)	3.9 (0.6)	4.0 (0.5)	3.8 (0.6)	3.8 (0.6)
Chair stand (s)	11.2 (3.6)	8.5 (1.3)	12.2 (2.8)	10.5 (2.8)
HGS (kg)	29.9 (8.7)	30.6 (8.3)	30.6 (5.9)	31.0 (6.5)
KES (Nm)	113 (50)	133 (42)	119 (46)	124 (39)
KES/AnT-MT (Nm/cm)	29.2 (11.9)	33.6 (9.5)	31.4 (11.2)	32.9 (8.8)

AnT MT, anterior thigh muscle thickness; HGS, handgrip strength; KES, knee extension strength.

All subjects were free from cardiovascular, metabolic, and immunologic disorders, as well as orthopedic abnormalities. Exclusion criteria were age younger than 50 years, taking any medication known to influence muscle mass and muscle function, and performing a regular high-intensity resistance training program. Participation in regular sports activity (at least twice a week and over the last 3 years) was assessed by a questionnaire. The study was conducted according to the World Medical Association Declaration of Helsinki and was approved by the institutional review board of the National Institute of Fitness and Sports in Kanoya. Written informed consent was obtained from all subjects before participation.

### Exercise intervention program

The subjects performed a 12-week body mass-based home exercise program, which aimed to improve the force-generating capabilities of the lower extremity muscles as described previously.^23^ Briefly, the circuit-type exercise program consisted of five exercises: (1) sitting down on and standing up from a chair, (2) standing hip joint extension (movement through the full range of motion), (3) standing side leg raises (same as above), (4) standing heel raises (same as above), and (5) trunk flexion in the seated position located in front of a chair (between upright abdominal crunch posture and semirecumbent position where their back contacted the back of a chair). The subjects were asked to perform the five exercises (one circuit) at a tempo of once every 2 sec, except trunk flexion/extension, which was completed in 4 sec.

The number of repetitions for each exercise was 16 repetitions per exercise (∼35 sec). Subjects were instructed to perform 2–3 circuits a day. All subjects conducted those circuits 3–6 days per week in their own home and once a week in a local gym as an exercise class. The subjects were requested to record the numbers of circuits they completed during each exercise session. The examiners then confirmed the number of circuits performed over a week. Within a short duration of resistance training, it is reported that resistance training-induced increases in strength are not affected by training volume (number of sets).^24^ Although the cross-education effect may be associated with exercise intensity,^20^ training-induced strength gains are assumed to be unaffected by weekly training volumes (number of circuits) in this study.

### Body composition and muscle thickness

Body composition was measured using an InBody 720 analyzer (Biospace Co. Ltd., Seoul, Korea) for subjects who participated in the Kanoya Chokin Study or Body Planner DF-800 (Yamato-Scale Co. Ltd., Akashi, Japan) for subjects who participated in the other two studies. The body composition analyzers adopt a tetrapolar, eight-point, tractile electrode system in both InBody 720 and Body Planner DF-800. The same analyzer was used for measuring body composition at both baseline and post-testing for each individual. Fat-free mass (FFM) was calculated as total body mass minus fat mass. Body mass and standing height were measured to the nearest 0.1 kg and 0.1 cm, respectively, by using an electronic weight scale and a stadiometer. Body–mass index (BMI) was defined as body mass/height^2^ (in kilograms per square meter).

Anterior thigh muscle thickness (MT) was measured using B-mode ultrasound (Aloka ProSound-2, Tokyo, Japan) at the anterior mid-thigh (at a distance between the lateral condyle of the femur and the greater trochanter) on the right side of the body as described previously.^25^ Briefly, the measurements were taken while the subjects stood quietly. A 7.5-MHz scanning head was placed on the skin surface of the measurement site using the minimum pressure required, and cross-sections of the muscle were imaged. Anterior thigh MT was measured as the perpendicular distance between the subcutaneous adipose tissue–muscle interface and the muscle–bone interface. Test-retest reliability of anterior thigh MT measurements, using the intraclass correlation coefficient (ICC_3,1_), standard error of measurement (SEM), and minimal difference, was previously determined for data from 15 middle-aged subjects scanned twice 24 h apart: 0.98, 0.07, and 0.19 cm, respectively.^26^

### Chair stand

The chair stand test required subjects to rise from a chair a total of 10 times as quickly as possible with arms placed across their chest. The elapsed time required to complete all 10 repetitions was recorded using a stopwatch (ADMD-001; Seiko, Tokyo, Japan) at baseline and after the intervention. Test-retest reliability of chair stand measurements, using ICC_3,1_, SEM, and minimal difference needed to be considered real, was previously determined for data from Japanese older adults (10 women and 4 men) tested twice within 2 days (0.967, 0.10, and 0.28 sec, respectively).

### Maximum strength measurements

HGS was measured for the right hand with a calibrated Smedley hand dynamometer (TKK 5401 Grip-D, Takei Scientific Instruments, Tokyo, Japan) before (baseline) and after (post) the intervention. All subjects were instructed to maintain an upright standing position with their arms placed down by their side, while holding the dynamometer without squeezing. The width of the dynamometer's handle was adjusted to the hand size of the subject (the middle phalanx rested on the inner handle). Subjects were allowed to perform one test trial, followed by two maximum trials, and the highest value was used for analysis. Test-retest reliability of HGS measurements, using ICC_3,1_, SEM, and minimal difference, was previously determined for data from the 23 subjects tested twice 24 h apart: 0.975, 2.5, and 7.0 kg, respectively.^27^

Maximum isometric KES was also measured for the right leg using a specially designed dynamometer (S-09010C, Takei Scientific Instruments, Tokyo, Japan) before and after the intervention. Subjects were seated on an adjustable chair and hip and knee joints were kept at 90° (a knee joint angle of zero corresponded to full extension of the knee). During the measurement, the hips and thighs were held tightly in the seat using adjustable lap belts. After a standardized warm-up consisting of stretching and submaximum contractions at ∼50% (two repetitions) and 80% (one repetition) of maximum effort, the subjects performed a maximum voluntary isometric contraction twice, with at least 2 min of rest between trials. If the difference between the torques of the two trials was more than 10%, the measurement was made again. The highest value was used for data analysis. Relative KES was calculated as KES/anterior thigh MT.

### Statistical analysis

A linear regression model adjusting for baseline covariates in a linear/nonlinear manner analyzed the impact that body mass-based lower body training had on HGS at post-testing, KES at post-testing, and anterior thigh MT at post-testing. To avoid multicollinearity issues, we clustered the variables to identify highly correlated variable clusters using generalized Spearman's rho. In that analysis, we discovered that KES, KES/body mass ratio, and KES/anterior thigh MT at baseline were clustered, so we decided to only include KES/body mass ratio in the regression models.

We also discovered that FFM and height were clustered and BMI and weight were clustered, so we only included FFM and BMI at baseline in our models. Because the intervention was likely to have less of an effect for stronger subjects and more of an effect for weaker subjects, we included an interaction between group and KES/body mass ratio. We used KES/body mass ratio as a surrogate for exercise intensity. Data were analyzed using R version 3.3.1^28^ and the RMS package.^29^

In addition, Pearson product correlations were performed to determine the relationships between HGS and KES in both baseline and post-testing. Statistical significance was set at *p* < 0.05.

## Results

Approximately three-quarters of the subjects (88 women and 37 men) reported participation in regular sports activities, including relatively slow walking on land or in water, calisthenics, and tai chi. The rate of regular sports activity was 81% in women and 71% in men. All sports activities were light intensity for the lower body, while the upper extremity muscles were not trained during these sports activities. The average training volume in the trained group was 14.8 (SD 8.3) circuits per week.

**FIG. 1.**
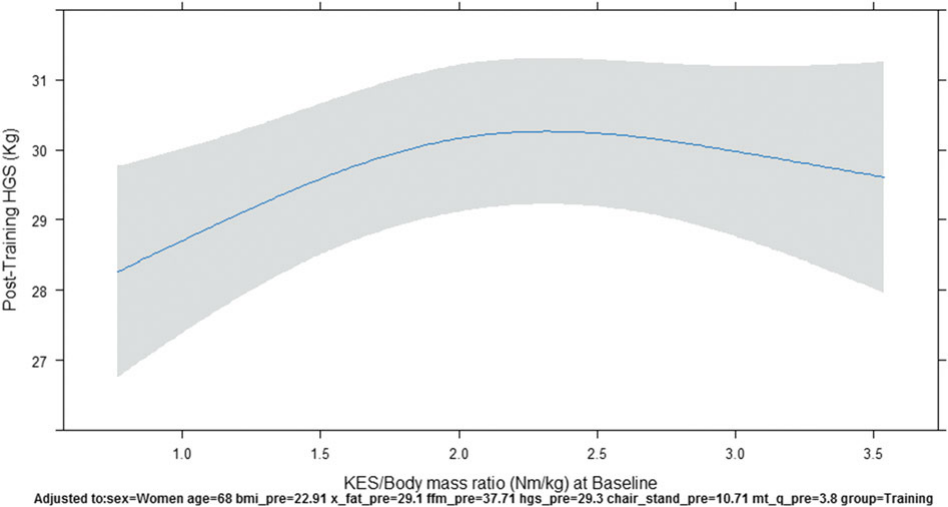
The plot KES/body mass ratio at baseline and HGS post-testing, controlling for baseline covariates. HGS, handgrip strength; KES, knee extension strength.

In our linear regression model after adjusting for baseline covariates, there was no statistically significant difference in post-testing HGS between groups (Control vs. Training) with the estimated difference (ED) between groups being −0.50 kg and the confidence interval (CI) being −1.56 to 0.55 kg. HGS at baseline was a statistically significant covariate with the expected difference from the 25th to 75th percentile being 8.39 kg with a CI of 7.48–9.30 kg. KES/body mass ratio at baseline was a statistically significant covariate with the expected difference from the 25th to 75th percentile being 0.76 kg with a CI of 0.04–1.49 kg. The plot of the nonlinear relationship of HGS-post and KES/body mass ratio is found in Figure 1. The overall fit of our model for post-testing HGS was good as we observed an adjusted R^2^ of 0.878 and a residual standard error of 2.77 kg.

After adjusting for baseline covariates, the linear regression model for post-testing KES found a statistically significant difference in post-testing KES between groups (Control vs. Training) with the ED being −12.02 Nm and a CI of −23.96 to −0.08 Nm. Furthermore, a statistically significant difference in post-testing KES was found between sexes with the ED 18.56 Nm and the CI being 4.99–32.13 Nm. Age was a statistically significant covariate with the expected difference between the 25th and 75th percentile being −4.67 Nm with a CI of −9.46 to 0.12 Nm. KES/body mass ratio at baseline was also a significant covariate with the ED between the 25th and 75th percentile being 27.91 Nm with a CI of 21.85–33.97 Nm. The overall fit of our model for post-testing KES was good as we observed an adjusted R^2^ of 0.74 with a residual standard error of 21.42 Nm.

After adjusting for baseline covariates, the linear regression model for post-testing anterior thigh MT found a statistically significant difference in post-testing anterior thigh MT between groups (Control vs. Training) with the ED being −0.13 cm and CI being −0.23 to −0.03 cm. Anterior thigh MT at baseline was also a significant covariate with the expected difference between the 25th and 75th percentiles being 0.51 cm and the CI being 0.44–0.58 cm. The overall fit of our model for post-testing anterior thigh MT was good as we observed an adjusted R^2^ of 0.777 with a residual standard error of 0.26 cm.

Supplementary Appendix A reports the expected change in outcome variables when increasing the independent covariate from the 25th to the 75th percentile when holding all other covariates constant. Further breakdown of the linear regression models can also be found in Supplementary Appendix A.

**FIG. 2.**
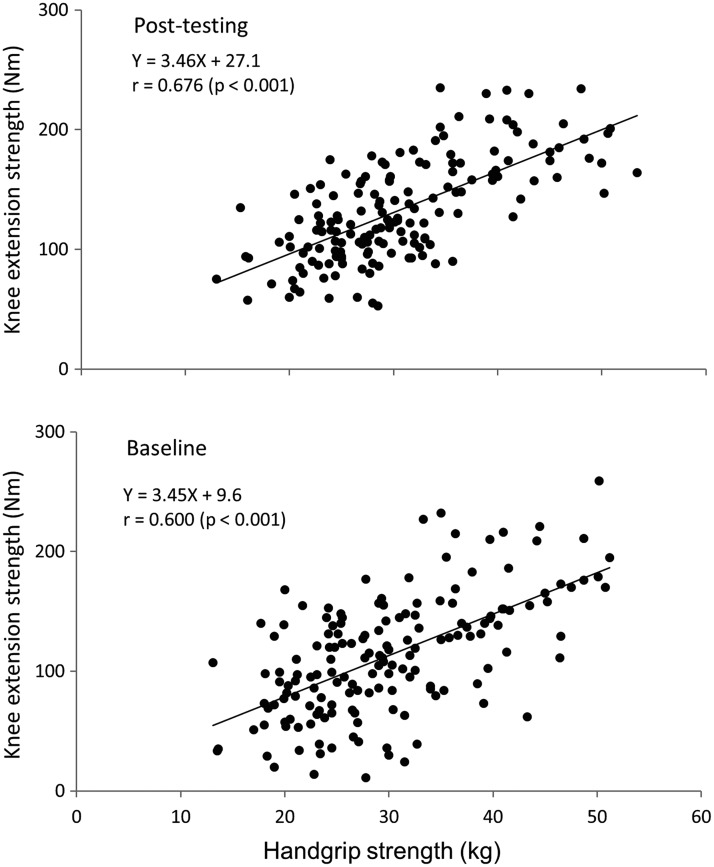
Relationships between HGS and isometric KES at baseline and post-testing in older adults.

There was a significant correlation between HGS and KES in both baseline (*r* = 0.600, *p* < 0.001) and post-testing (*r* = 0.676, *p* < 0.001) for the training group (Fig. 2) and between HGS and KES in both baseline (*r* = 0.32, *p* < 0.05) and post-testing (*r* = 0.43, *p* < 0.01) for the control group. In the training group, the slope of the regression line was identical between baseline and post-testing, while the value of the y-intercept was shifted from 9.6 Nm in baseline to 27.1 Nm in post-testing (Fig. 2).

## Discussion

The main findings of the present study were that (1) lower extremity muscular function and anterior thigh MT significantly improved more in a training group that performed 12 weeks of lower body mass-based exercise than a control group; however, (2) a cross-education effect for improvements in HGS was not observed.

### Effects of intervention on lower body strength and muscle size

In the present study, a significant difference between the training group and control group was found for isometric KES and anterior thigh MT. The average change for isometric KES was 18%, while the average change in anterior thigh MT was 3% (Table 1). The magnitude of increase in those variables is similar to a previous study (isometric KES increased by 14%) investigating the effects of a body mass-based intervention on strength in middle-aged and older women.^23^ However, our results are relatively low compared with a previous study (dynamic KES increased by 23% and anterior thigh MT increased by 7%) that had young and middle-aged adults perform 12 weeks of high-intensity resistance training.^30^

It has been hypothesized that high-intensity dynamic resistance training induces higher mechanical stress and less metabolic fatigue than low-intensity resistance training when exercise is performed until failure.^31^ In contrast, low-intensity dynamic resistance training produces less mechanical stress, but higher metabolic fatigue.^31^ Both mechanical stress and metabolic fatigue may contribute to training-induced muscle growth and improved strength.^32^ Exercise intensity (i.e., percentage of the maximal electromyographic activity [%EMG max]) in the quadriceps during a body mass-based squat exercise depends on a KES/body mass ratio. The %EMG max is nonlinearly related to KES/body mass ratio with a breakpoint of nonlinear regression found at 1.9 Nm/kg body mass.^22^ In the present study, the average KES/body mass ratio was 2.0 Nm/kg, which may correspond approximately to 40%EMG max during the squat exercise.

**FIG. 3.**
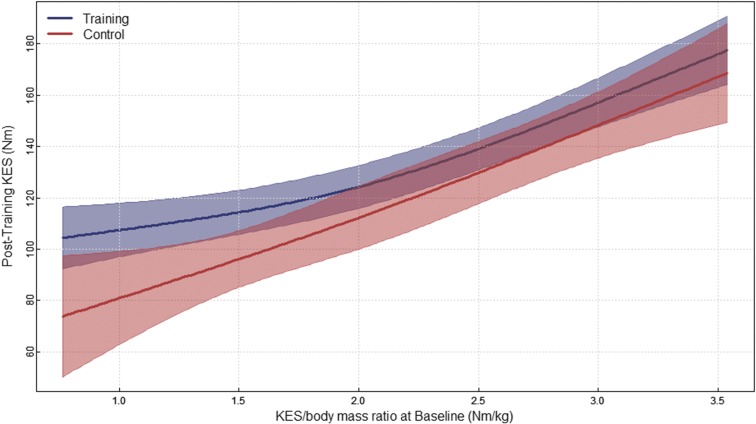
The plot of KES/body mass ratio at baseline and KES post-testing, controlling for baseline covariates separated by group.

It is expected that individuals with lower strength would be exercising at a higher relative intensity throughout the training compared with individuals with greater strength who would be working at a lower relative exercise intensity.^22^ In the current study, as individual's baseline relative strength (KES/body mass ratio) increased, KES at post-testing increased. When examining the plot of differences in groups for post-KES (Fig. 3), increasing baseline relative strength resulted in increases in post-KES with the training group having higher KES values than the control group. It appears from the plot (Fig. 3) that the difference in groups is greater in individuals with lower relative strength, but a statistical interaction effect was not found. This leads to the idea that individuals with low relative strength may be working at higher relative intensities, which can produce greater changes in strength, but this is inconclusive from our data set and should be confirmed in future studies.

### Effects of intervention on HGS

A study investigating the effects of high-intensity (75–80% of 1RM), machine-based, whole body resistance training reported that KES increased (*p* < 0.001) by 58 N (12%) and HGS increased (*p* < 0.001) by 1.7 kg (6%) in healthy older adults (*n* = 198).^11^ As described above, high-intensity, machine-based resistance training may involve the indirect handgrip exercise effect. At the start of the present study, our hypothesis was that HGS would be improved through training-induced increases in lower body muscular strength in the absence of direct and indirect handgrip exercise.

In contradiction to our hypothesis, however, no significant change in HGS was observed following 12 weeks of body mass-based lower body exercise training even for subjects who had a low KES/body mass ratio. For individuals with strength less than 1.9 Nm/kg, the average KES/body mass ratio was 1.3 Nm/kg and the estimated exercise intensity during a body mass-based squat exercise would be ∼55%EMG max.^22^ Therefore, it is expected that a cross-education effect may appear between trained unilateral and untrained contralateral arms or legs when using similar exercise intensity.^19,20^

Despite a sufficient intensity to potentially promote a cross-education effect across contralateral legs, a cross-education effect was not found for improvements in HGS when comparing HGS in the training and control groups. Interestingly, a significant nonlinear relationship was found between relative strength (KES/body mass ratio) at baseline and HGS post-testing. As baseline relative strength increased, post-HGS increased, but when baseline relative strength went above ∼2.0 Nm/kg, post-HGS plateaued. The lack of increase in HGS despite increases in relative strength may be due to already present neurological adaptations found in individuals with greater relative strength.

In addition, it is known that the cross-education effect is not age or sex specific.^19^ Similarly, the cross-education effect is not specifically observed in only one muscle group.^19^ As mentioned above, our results showed that KES increased following body mass-based training, but this increase was relatively low compared with a previous study.^30^ Performing resistance exercise, followed by intake of sufficient protein, results in an augmentation of muscle protein synthesis, which can lead to muscle hypertrophy and strength gain.^33^ Unfortunately, we did not measure nutritional status of our subjects before and during the intervention. Future research should investigate those possibilities.

In conclusion, the present study tested if improvements in lower body strength from nonmachine body-strengthening exercises would cross over to HGS in older adults. Our results suggest that lower body increases in strength and muscle size following a 12-week lower body mass-based exercise intervention fail to translate into improvements in HGS.

## Supplementary Material

Supplemental data

## Abbreviations Used

BMI: body–mass index
CI: confidence interval
ED: estimated difference
FFM: fat-free mass
HGS: handgrip strength
KES: knee extension strength
MT: muscle thickness
SD: standard deviation
SEM: standard error of measurement
