# Supplementary material for: Can Handgrip Strength Improve Following Body Mass-Based Lower Body Exercise?
Source: Biores Open Access. 2017 Mar 1;6(1):19–27. doi: 10.1089/biores.2017.0008 (PMC5385419; doi:10.1089/biores.2017.0008)
Supplement: Supplemental data [file Supp_AppendixA.doc]

# Appendix A

Table of Contents

[DATA 1](#__RefHeading___Toc462257346)

[DESCRIPTIVE TABLE 2](#__RefHeading___Toc462257347)

[VARIABLE CLUSTERS 3](#__RefHeading___Toc462257348)

[REGRESSION ANALYSIS HGS POST-TRAINING 4](#__RefHeading___Toc462257349)

[REGRESSION ANALYSIS KES POST-TRAINING 6](#__RefHeading___Toc462257350)

[REGRESSION ANALYSIS AnT-MT POST-TRAINING 9](#__RefHeading___Toc462257351)

## DATA

The data for this analysis is from 197 participants, collected from three cohort studies: the Kanoya Chokin Study (Yoshitake et al. 2011), Shibushi Chokin Study, and Bando Chokin Study.

The following table provides descriptive statistics for the baseline and follow-up data collected in this study.

## DESCRIPTIVE TABLE

|  | N | Control | Training |
| --- | --- | --- | --- |
|  |  | (N=37) | (N=160) |
| Sex : Women | 197 | 0.57 ( 21) | 0.68 (108) |
| Age | 197 | 65/69/72 | 65/68/72 |
| Height Pre (cm) | 197 | 152/156/159 | 150/154/160 |
| Height Post (cm) | 197 | 152/156/159 | 150/155/161 |
| Weight Pre (kg) | 197 | 49.4/56.0/61.4 | 50.1/55.2/60.5 |
| Weight Post (kg) | 197 | 49.0/56.3/61.5 | 50.0/55.0/60.5 |
| Body Mass Index Pre (kg/m2) | 197 | 20.8/22.9/24.4 | 21.1/22.9/24.9 |
| Body Mass Index Post (kg/m2) | 197 | 21.0/22.6/24.1 | 21.0/22.8/24.6 |
| Body fat % Pre | 197 | 25.2/29.1/32.5 | 24.7/29.1/33.6 |
| Body fat % Post | 197 | 24.5/28.7/33.8 | 24.2/28.8/33.8 |
| Fat-Free Mass Pre (kg) | 197 | 35.6/39.1/43.5 | 34.7/37.5/44.2 |
| Fat-Free Mass Post (kg) | 197 | 35.3/39.1/46.0 | 34.4/37.4/44.2 |
| HGS pre (kg) | 197 | 27.4/30.5/35.0 | 23.4/29.0/35.6 |
| Handgrip Strength Post (cm) | 197 | 27.3/31.0/34.6 | 24.5/29.1/35.3 |
| Chairstand Time Pre (s) | 196 | 9.5/11.9/14.3 | 9.3/10.3/12.1 |
| Chairstand Time Post (s) | 196 | 8.90/ 9.90/11.26 | 7.73/ 8.30/ 9.07 |
| Knee-Extension Strength (KES) Pre (Nm) | 197 | 86.0/108.0/150.4 | 77.8/111.0/145.2 |
| Knee-Extension Strength (KES) Post (Nm) | 197 | 96.1/118.0/153.0 | 101.5/125.5/163.0 |
| Anterior Thigh Muscle Thickness (MT) Pre (cm) | 197 | 3.40/3.80/4.10 | 3.49/3.85/4.20 |
| Anterior Thigh Muscle Thickness (MT)Post (cm) | 197 | 3.30/3.85/4.07 | 3.60/3.90/4.27 |
| KES/Anterior thigh MT Pre (Nm/cm) | 197 | 24.5/29.5/41.4 | 20.9/28.9/37.1 |
| KES/Anterior thigh MT Post (Nm/cm) | 197 | 25.4/32.9/39.7 | 26.9/32.8/40.1 |
| KES/Body Mass Ratio Pre (Nm/kg) | 197 | 1.46/1.94/2.83 | 1.40/2.01/2.54 |
| KES/Body Mass Ratio Post (Nm/kg) | 197 | 1.71/2.23/2.71 | 1.91/2.33/2.82 |

## VARIABLE CLUSTERS

Kes_mt_pre = Knee extension/anterior thigh muscle thickness pre, kes_bm_pre = knee extension/body mass ratio pre, kes_pre = knee extension strength pre, x_fat_pre = body fat % pre, hgs_pre = handgrip strength pre, ffm_pre = fat-free mass pre, ht_pre = height pre, mt_q_pre= anterior thigh muscle thickness pre, bmi_pre = body mass index pre, wt_pre = weight pre.


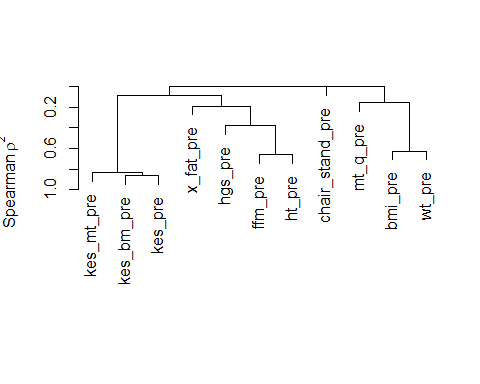


## REGRESSION ANALYSIS HGS POST-TRAINING

|  | d.f. | Partial SS | MS | F | P |  | |
| --- | --- | --- | --- | --- | --- | --- | --- |
| Sex | 1 | 12.30 | 12.30 | 1.60 | 0.21 |  | |
| Age | 2 | 14.55 | 7.27 | 0.95 | 0.39 |  | |
| Nonlinear | 1 | 1.62 | 1.62 | 0.21 | 0.65 |  | |
| BMI pre | 2 | 39.27 | 19.64 | 2.55 | 0.08 |  | |
| Nonlinear | 1 | 29.64 | 29.64 | 3.85 | 0.05 |  | |
| Body fat % pre | 2 | 4.55 | 2.28 | 0.30 | 0.74 |  | |
| Nonlinear | 1 | 2.45 | 2.45 | 0.32 | 0.57 |  | |
| Fat-free mass pre | 2 | 29.78 | 14.89 | 1.94 | 0.15 |  | |
| Nonlinear | 1 | 6.34 | 6.34 | 0.82 | 0.37 |  | |
| HGS pre | 2 | 2657.00 | 1328.50 | 172.74 | 0.00 |  | |
| Nonlinear | 1 | 5.39 | 5.39 | 0.70 | 0.40 |  | |
| Chair Stand pre | 2 | 7.01 | 3.51 | 0.46 | 0.63 |  | |
| Nonlinear | 1 | 6.08 | 6.08 | 0.79 | 0.38 |  | |
| Anterior thigh MT pre | 2 | 23.71 | 11.85 | 1.54 | 0.22 |  | |
| Nonlinear | 1 | 6.25 | 6.25 | 0.81 | 0.37 |  | |
| KES/body mass ratio pre | 2 | 66.03 | 33.02 | 4.29 | 0.02 |  | |
| Nonlinear | 1 | 42.12 | 42.12 | 5.48 | 0.02 |  | |
| group | 1 | 6.76 | 6.76 | 0.88 | 0.35 |  | |
| TOTAL NONLINEAR | 8 | 95.84 | 11.98 | 1.56 | 0.14 |  | |
| TOTAL | 18 | 10890.04 | 605.00 | 78.67 | 0.00 |  | |
| ERROR | 177 | 1361.25 | 7.69 | NA | NA |  | |
|  |  |  |  |  |  |  | |
|  | model$stats |  | | | | | |
| n | 196.00 |  | | | | | |
| Model L.R. | 430.66 |  | | | | | |
| d.f. | 18.00 |  | | | | | |
| R2 | 0.89 |  | | | | | |
| g | 8.43 |  | | | | | |
| Sigma | 2.77 |  | | | | | |
|  |  |  | | | | | |
|  |  |  | | | | | |
|  | Low | High | Diff. | Effect | S.E. | Lower 0.95 | Upper 0.95 |
| Age | 65.00 | 72.00 | 7.00 | -0.41 | 0.31 | -1.02 | 0.21 |
| BMI pre | 21.01 | 24.86 | 3.85 | -0.27 | 0.78 | -1.81 | 1.26 |
| Body fat % pre | 24.70 | 33.40 | 8.70 | 0.41 | 0.71 | -0.99 | 1.81 |
| Fat-free mass pre | 34.83 | 44.18 | 9.35 | 1.63 | 0.83 | -0.01 | 3.26 |
| HGS pre | 23.90 | 35.50 | 11.60 | 8.39 | 0.46 | 7.48 | 9.30 |
| Chair Stand pre | 9.38 | 12.60 | 3.22 | 0.37 | 0.40 | -0.41 | 1.15 |
| Anterior thigh MT pre | 3.45 | 4.20 | 0.75 | 0.45 | 0.37 | -0.27 | 1.17 |
| KES/body mass ratio pre | 1.42 | 2.56 | 1.14 | 0.76 | 0.37 | 0.04 | 1.49 |
| Sex - Men:Women | 2.00 | 1.00 | NA | 1.11 | 0.88 | -0.62 | 2.83 |
| group - Control:Training | 2.00 | 1.00 | NA | -0.50 | 0.54 | -1.56 | 0.55 |


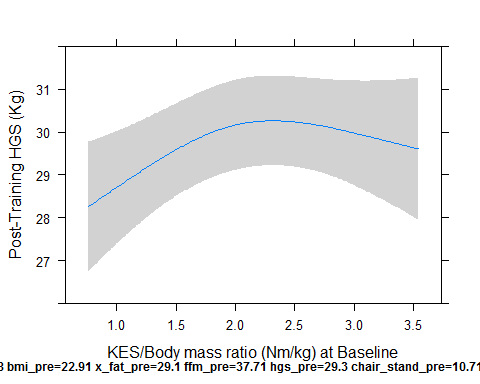


## REGRESSION ANALYSIS KES POST-TRAINING

|  | d.f. | Partial SS | MS | F | P |  | |
| --- | --- | --- | --- | --- | --- | --- | --- |
| Sex | 1 | 3345.14 | 3345.14 | 7.29 | 0.01 |  | |
| Age | 2 | 3893.75 | 1946.88 | 4.24 | 0.02 |  | |
| Nonlinear | 1 | 2124.43 | 2124.43 | 4.63 | 0.03 |  | |
| BMI pre | 2 | 1593.80 | 796.90 | 1.74 | 0.18 |  | |
| Nonlinear | 1 | 502.13 | 502.13 | 1.09 | 0.30 |  | |
| Body fat % pre | 2 | 3340.79 | 1670.39 | 3.64 | 0.03 |  | |
| Nonlinear | 1 | 175.55 | 175.55 | 0.38 | 0.54 |  | |
| Fat-free mass pre | 2 | 6556.27 | 3278.13 | 7.14 | 0.00 |  | |
| Nonlinear | 1 | 806.01 | 806.01 | 1.76 | 0.19 |  | |
| HGS pre | 2 | 822.25 | 411.12 | 0.90 | 0.41 |  | |
| Nonlinear | 1 | 111.43 | 111.43 | 0.24 | 0.62 |  | |
| Chair Stand pre | 2 | 1041.81 | 520.91 | 1.14 | 0.32 |  | |
| Nonlinear | 1 | 31.75 | 31.75 | 0.07 | 0.79 |  | |
| Anterior thigh MT pre | 2 | 514.51 | 257.25 | 0.56 | 0.57 |  | |
| Nonlinear | 1 | 92.62 | 92.62 | 0.20 | 0.65 |  | |
| KES/body mass ratio pre (Factor+Higher Order Factors) | 4 | 59434.40 | 14858.60 | 32.39 | 0.00 |  | |
| All Interactions | 2 | 1055.70 | 527.85 | 1.15 | 0.32 |  | |
| Nonlinear (Factor+Higher Order Factors) | 2 | 3394.67 | 1697.34 | 3.70 | 0.03 |  | |
| group (Factor+Higher Order Factors) | 3 | 6806.10 | 2268.70 | 4.94 | 0.00 |  | |
| All Interactions | 2 | 1055.70 | 527.85 | 1.15 | 0.32 |  | |
| KES/body mass ratio pre * group (Factor+Higher Order Factors) | 2 | 1055.70 | 527.85 | 1.15 | 0.32 |  | |
| Nonlinear | 1 | 259.79 | 259.79 | 0.57 | 0.45 |  | |
| Nonlinear Interaction : f(A,B) vs. AB | 1 | 259.79 | 259.79 | 0.57 | 0.45 |  | |
| TOTAL NONLINEAR | 9 | 8198.17 | 910.91 | 1.99 | 0.04 |  | |
| TOTAL NONLINEAR + INTERACTION | 10 | 9452.35 | 945.23 | 2.06 | 0.03 |  | |
| TOTAL | 20 | 257763.46 | 12888.17 | 28.09 | 0.00 |  | |
| ERROR | 175 | 80291.85 | 458.81 | NA | NA |  | |
|  |  |  |  |  |  |  | |
|  | model$stats |  | | | | | |
| n | 196.00 |  | | | | | |
| Model L.R. | 281.76 |  | | | | | |
| d.f. | 20.00 |  | | | | | |
| R2 | 0.76 |  | | | | | |
| g | 41.08 |  | | | | | |
| Sigma | 21.42 |  | | | | | |
|  | Low | High | Diff. | Effect | S.E. | Lower 0.95 | Upper 0.95 |
| Age | 65.00 | 72.00 | 7.00 | -4.67 | 2.43 | -9.46 | 0.12 |
| BMI pre | 21.01 | 24.86 | 3.85 | -6.52 | 6.02 | -18.40 | 5.35 |
| Body fat % pre | 24.70 | 33.40 | 8.70 | 14.74 | 5.51 | 3.86 | 25.62 |
| Fat-free mass pre | 34.83 | 44.18 | 9.35 | 24.30 | 6.44 | 11.60 | 37.01 |
| HGS pre | 23.90 | 35.50 | 11.60 | 4.19 | 3.62 | -2.95 | 11.34 |
| Chair Stand pre | 9.38 | 12.60 | 3.22 | -1.84 | 3.07 | -7.91 | 4.22 |
| Anterior thigh MT pre | 3.45 | 4.20 | 0.75 | 2.95 | 2.83 | -2.64 | 8.53 |
| KES/body mass ratio pre | 1.42 | 2.56 | 1.14 | 27.91 | 3.07 | 21.85 | 33.97 |
| Sex - Men:Women | 2.00 | 1.00 | NA | 18.56 | 6.87 | 4.99 | 32.13 |
| group - Control:Training | 2.00 | 1.00 | NA | -12.02 | 6.05 | -23.96 | -0.08 |


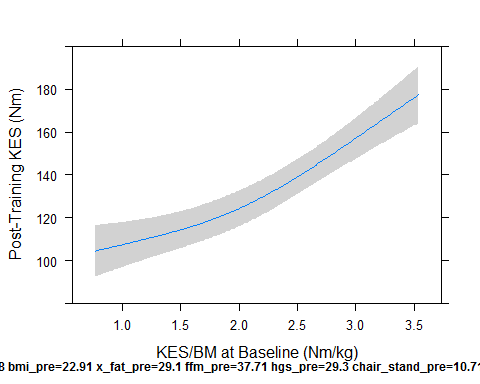


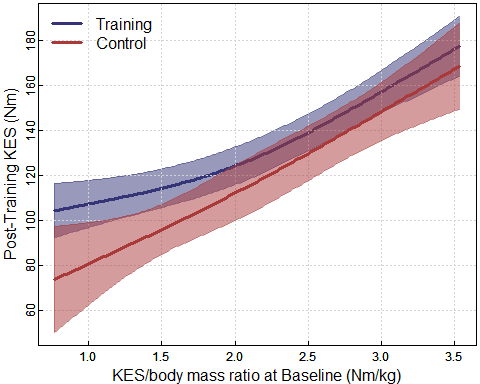


## REGRESSION ANALYSIS ANTERIOR THIGH-MT POST-TRAINING

|  | d.f. | Partial SS | MS | F | P |  | |
| --- | --- | --- | --- | --- | --- | --- | --- |
| Sex | 1 | 0.05 | 0.05 | 0.76 | 0.39 |  | |
| Age | 2 | 0.28 | 0.14 | 2.05 | 0.13 |  | |
| Nonlinear | 1 | 0.16 | 0.16 | 2.36 | 0.13 |  | |
| BMI pre | 2 | 0.21 | 0.10 | 1.49 | 0.23 |  | |
| Nonlinear | 1 | 0.17 | 0.17 | 2.42 | 0.12 |  | |
| Body fat % pre | 2 | 0.31 | 0.16 | 2.28 | 0.10 |  | |
| Nonlinear | 1 | 0.28 | 0.28 | 4.00 | 0.05 |  | |
| Fat-free mass pre | 2 | 0.01 | 0.01 | 0.11 | 0.90 |  | |
| Nonlinear | 1 | 0.00 | 0.00 | 0.00 | 0.98 |  | |
| HGS pre | 2 | 0.07 | 0.04 | 0.54 | 0.59 |  | |
| Nonlinear | 1 | 0.04 | 0.04 | 0.62 | 0.43 |  | |
| Chair Stand pre | 2 | 0.36 | 0.18 | 2.60 | 0.08 |  | |
| Nonlinear | 1 | 0.07 | 0.07 | 1.04 | 0.31 |  | |
| Anterior thigh MT pre | 2 | 15.52 | 7.76 | 112.58 | 0.00 |  | |
| Nonlinear | 1 | 0.11 | 0.11 | 1.63 | 0.20 |  | |
| KES/body mass ratio pre | 2 | 0.38 | 0.19 | 2.75 | 0.07 |  | |
| Nonlinear | 1 | 0.19 | 0.19 | 2.74 | 0.10 |  | |
| group | 1 | 0.48 | 0.48 | 6.99 | 0.01 |  | |
| TOTAL NONLINEAR | 8 | 1.10 | 0.14 | 1.99 | 0.05 |  | |
| TOTAL | 18 | 47.95 | 2.66 | 38.65 | 0.00 |  | |
| ERROR | 177 | 12.20 | 0.07 | NA | NA |  | |
|  | model$stats |  | | | | | |
| n | 196.00 |  | | | | | |
| Model L.R. | 312.69 |  | | | | | |
| d.f. | 18.00 |  | | | | | |
| R2 | 0.80 |  | | | | | |
| g | 0.56 |  | | | | | |
| Sigma | 0.26 |  | | | | | |
|  |  |  | | | | | |
|  | Low | High | Diff. | Effect | S.E. | Lower 0.95 | Upper 0.95 |
| Age | 65.00 | 72.00 | 7.00 | -0.04 | 0.03 | -0.10 | 0.02 |
| BMI pre | 21.01 | 24.86 | 3.85 | 0.09 | 0.07 | -0.05 | 0.24 |
| Body fat % pre | 24.70 | 33.40 | 8.70 | -0.04 | 0.07 | -0.17 | 0.10 |
| Fat-free mass pre | 34.83 | 44.18 | 9.35 | 0.03 | 0.08 | -0.12 | 0.19 |
| HGS pre | 23.90 | 35.50 | 11.60 | -0.02 | 0.04 | -0.11 | 0.06 |
| Chair Stand pre | 9.38 | 12.60 | 3.22 | -0.07 | 0.04 | -0.15 | 0.00 |
| Anterior thigh MT pre | 3.45 | 4.20 | 0.75 | 0.51 | 0.03 | 0.44 | 0.58 |
| KES/body mass ratio pre | 1.42 | 2.56 | 1.14 | 0.05 | 0.03 | -0.02 | 0.12 |
| Sex - Men:Women | 2.00 | 1.00 | NA | 0.07 | 0.08 | -0.09 | 0.24 |
| group - Control:Training | 2.00 | 1.00 | NA | -0.13 | 0.05 | -0.23 | -0.03 |
